# Photo-Polymerization Damage Protection by Hydrogen Sulfide Donors for 3D-Cell Culture Systems Optimization

**DOI:** 10.3390/ijms22116095

**Published:** 2021-06-05

**Authors:** Silvia Buonvino, Matteo Ciocci, Dror Seliktar, Sonia Melino

**Affiliations:** 1 Department of Chemical Science and Technologies, University of Rome “Tor Vergata”, 00133 Rome, Italy; silvia.buonvino95@gmail.com (S.B.); ciocci.matteo@gmail.com (M.C.); 2 Department of Biomedical Engineering, Technion-Israel Institute of Technology, Haifa 3200003, Israel; bmdror@technion.ac.il; 3 Russell Berrie Nanotechnology Institute (RBNI), Technion-Israel Institute of Technology, Haifa 3200003, Israel; 4 Center for Regenerative Medicine CIMER, University of Rome “Tor Vergata”, 00133 Rome, Italy

**Keywords:** photo-polymerization, protein hydrogel, tissue repair, stem cells, enzyme, radicals

## Abstract

Photo-polymerized hydrogels are ideally suited for stem-cell based tissue regeneration and three dimensional (3D) bioprinting because they can be highly biocompatible, injectable, easy to use, and their mechanical and physical properties can be controlled. However, photo-polymerization involves the use of potentially toxic photo-initiators, exposure to ultraviolet light radiation, formation of free radicals that trigger the cross-linking reaction, and other events whose effects on cells are not yet fully understood. The purpose of this study was to examine the effects of hydrogen sulfide (H_2_S) in mitigating cellular toxicity of photo-polymerization caused to resident cells during the process of hydrogel formation. H_2_S, which is the latest discovered member of the *gasotransmitter* family of gaseous signalling molecules, has a number of established beneficial properties, including cell protection from oxidative damage both directly (by acting as a scavenger molecule) and indirectly (by inducing the expression of anti-oxidant proteins in the cell). Cells were exposed to slow release H_2_S treatment using pre-conditioning with glutathione-conjugated-garlic extract in order to mitigate toxicity during the photo-polymerization process of hydrogel formation. The protective effects of the H_2_S treatment were evaluated in both an enzymatic model and a 3D cell culture system using cell viability as a quantitative indicator. The protective effect of H_2_S treatment of cells is a promising approach to enhance cell survival in tissue engineering applications requiring photo-polymerized hydrogel scaffolds.

## 1. Introduction

**Figure 1:**
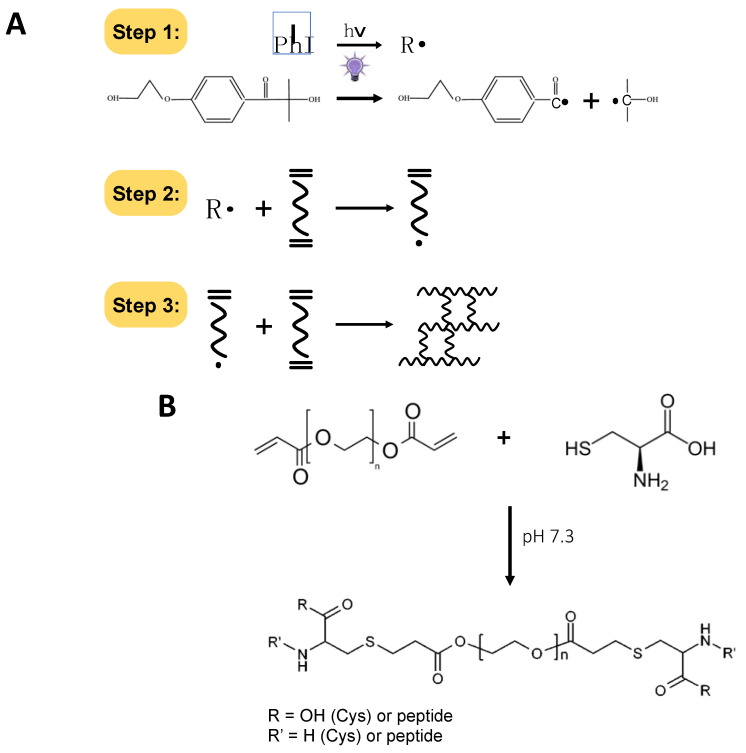
Schematic description of chemical reactions linked to the PhP damage. (**A**) Scheme of the reaction of free-radical formation from the photo-initiator (I) (step 1) leading to the photo-polymerization of the hydrogel by cross-linking (steps 2 and 3); (**B**) scheme of the Michael-type addition reaction to form the ester bond between the acrylate end-groups on PEGDA and the free thiols in the protein cysteines.

Stem-cell based therapy represents an innovative approach for the repair and regeneration of injured organs and tissues. Stem-cells (SCs) are a source of undifferentiated cells, able to actively migrate to the site of injury and replace the damaged cells to reconstitute a functioning tissue [1,2]. Some companies are currently conducting clinical trials to treat diseases using bone marrow/adipose-derived mesenchymal stem cells (MSC) and several products are in phase II and III trials [3,4]. Preclinical studies have shown that the bulk of cells transplanted, regardless of cell type, fails to engraft, likely due to a combination of washout and a hostile environment, potentially limiting efficacy [5]. This happens because cells encounter a hostile environment, characterized by inflammation, cytokines, hypoxic conditions, a high concentration of reactive oxygen species (ROS), and other harmful features [6]. Usually less than 10% of the implanted cells survive the engraftment at the site of injury within days of implantation [7,8]. This limitation has spurred biomaterial and tissue-engineering efforts to enhance cell retention and survival [9]. Towards this aim, much progress has been made in the fields of encapsulating, injectable biomaterials as bio-scaffolds. These cell-laden scaffolds, namely, hydrogels, have produced promising experimental outcomes in cell therapies, revealing themselves to be key components not only as cell delivery vehicles or protective mechanical supports that enhance cell retention and survival during tissue regeneration, but also instructive systems that improve the efficacy of the engraftment [10]. Hydrogels are 3D polymeric networks consisting of cross-linked hydrophilic polymers that display unique characteristics, including a high degree of swelling when in contact with water. Hydrogels are promising materials for cell delivery because their highly hydrated nature mimics the basic properties of native tissues. Hydrogels are also readily functionalized with biological motifs that can modulate cell adhesion, proliferation and differentiation, making them ideal materials for use in the construction of bioactive stem cell carrier systems [11]. These materials have been exploited in many biomedical fields owing to their excellent biocompatibility and high permeability to oxygen and nutrients [12], which is critically important for cell encapsulation [13]. Many hydrogel scaffolds are also injectable, and their *sol-gel* transition can be carried out under mild condition in the presence of living cells [14]. This approach allows uniform cell seeding into the scaffold during the in situ formation of the hydrogel through minimal invasive methods that use catheters and laparoscopic devices. Importantly, the injectable hydrogels can help to keep the cells in the site of interest, while providing the protective features of an encapsulating environment. This protection prevents anoikis, a form of programmed cell death that occurs in anchorage-dependent cells when they detach from the surrounding extracellular matrix (ECM) [15]. Among the many methods used to cross-link the hydrogels, photo-polymerization is unique in that it provides both spatial and temporal control over the in situ *sol-gel* transition [16,17]. Photo-polymerization of hydrogels is considered a rapid curing mechanism, despite the fact that it uses low initiation light energies. The initiation can be applied at room temperature and does not require toxic organic solvents [18]. Many light-activated cross-linking reactions involve the use of a long-range UV light (i.e., UVA, 320–400 nm) in the presence of a photo-initiator that generates free-radicals. The initiator’s chemical nature will determine its interaction with the UVA radiation, the radical formation rate, and the specific wavelength of absorption. The free radicals in turn will react with functional groups on the polymer backbone, forming intermolecular bonds (Figure 1A). Most biopolymers must be chemically modified to be photo-cross-linkable, typically by introducing functional groups such as the widely used modification of poly(ethylene)glycol diacrylates (PEGDA) [19,20,21]. Free radicals produced during hydrogel assembly can induce unwanted cellular damage by directly reacting with cellular components, including proteins, or by forming reactive oxygen species (ROS) [22]. Furthermore, the Michael-type addition reaction (Figure 1B) could also cause unwanted reactions between the synthetic polymer and the cysteines of cell-membrane proteins when the cells are embedded in PEG-protein hydrogel-based scaffolds. Usually, a good cell survival 3D system with encapsulated cells is obtained using a precursor hydrogel solution with a very high cell density, ranging from 2 to 5 × 10^6^ cells/mL and in some cases 3 × 10^7^cells/mL [23,24,25,26]. This cellular density is very far from the cellular density used for cell growth on a plate. Thus, despite that several polymers and photo-initiators were demonstrated to exhibit a good toxicological profile [27], detailed studies about the effects of photo-polymerization on the embedded cells are needed.

### Hydrogen Sulfide-Releasing Agents to Prevent Oxidative Damage

One approach to mitigate cell damage associated with photo-polymerization has been to use antioxidants during the reaction. Antioxidant molecules, such as ascorbate, have been proven to significantly reduce the harmful effects of the free radicals on the cells during exposure to photo-initiation, particularly when cells were cultured on tissue culture plates (TCPs) [28]. However, antioxidants are also radical scavengers and therefore can reduce or inhibit the photo-polymerization reaction. For the reasons stated above, other protective molecules are sought to mitigate cellular toxicity of photo-polymerization without altering the cross-link reaction. During the past decade, the effects of the *gasotransmitter* H_2_S on SCs with different tissue origins have been investigated both in vitro and in vivo through different experimental approaches. Exogenous H_2_S can have both pro- [29,30,31,32] or anti-apoptotic effects [33,34,35,36] depending on the individual cell phenotype and on the experimental settings used (e.g., H_2_S concentration). Previous studies suggest that garlic-derived H_2_S- donors selectively induce programmed cell death in neoplastic cells, but not in their physiological counterparts or in adult SCs [27,28,29,30,31,32,33,34,35,36,37,38,39,40,41,42,43,44]. H_2_S has demonstrated an ability to improve cell survival by activation of multiple molecular signaling pathways [45] and may play a central role in the regulation of homeostasis and stemness of MSC [46]. Importantly, engrafted MSC preconditioned with H_2_S were able to enhance cardiac repair following myocardial infarction in rats [47]. Moreover, preconditioning of cardiac MSC (cMSC) and bone marrow MSC (BM-MSC) with exogenous H_2_S resulted in an improvement of their therapeutic potential by promoting proliferation and suppressing apoptosis under hypoxia-ischemic conditions [48,49]. Accordingly, H_2_S preconditioning of SCs may ameliorate the survival and efficacy of scaffold-embedded SCs for tissue repair and regeneration. In this context, two cell types—normal human dermal fibroblast (NHDF) and Sca-1^+^ Lin^-^ human cMSC—were preconditioned with H_2_S-releasing garlic extract (GSGa) [30,49], suspended in a hydrogel precursor solution, and exposed to photo-polymerization damage (PhP damage) of the cross-linking reaction. A PEG-fibrinogen hydrogel (PFHy) [11,20,50] was used here as encapsulating photo-polymerizable biomaterial. In this hydrogel, the fibrinogen biofunctionality and the structural versatility of the PEG molecules are combined and the stiffness can be easily modulated by increasing the cross-linking density. Therefore, it represents a good model of a photo-polymerizable hydrogel due to its prevalence in tissue engineering applications and because of its potential as a bio-ink in 3D bioprinting [26,50].

The PhP damage on the cellular proteins containing cysteine residues was also investigated by analysing the retained activity of a model enzyme: the thiosulfate: cyanide sulfurtransferase (TST, also named rhodanese) from *Azotobacter vinelandii* [51]. This recombinant cellular detoxification enzyme is characterized by the presence of only one cysteine residue located in the active site. We demonstrated that the damage from radicals on proteins and cells could be significantly inhibited by the presence of H_2_S-releasing donors and the preconditioning of MSC with H_2_S donors, such as GSGa, before the embedding in the photo-polymerizable hydrogel, which can improve their survival and proliferation in the carrier system.

## 2. Results and Discussion

### 2.1. A Model Enzyme for Studying PhP-Damage

The TST enzyme is a widely distributed sulfurtransferase, which catalyzes the in vitro production of thiocyanate, transferring the sulfane sulfur atom from thiosulfate to cyanide, by a double displacement mechanism (ping-pong reaction). In particular, the enzyme cycles between two catalytic intermediates: the sulfur un-loaded (E) and the sulfur loaded form (ES) that is characteristic of a persulfide (S-S) bond between the cysteine and the sulfur derived from a donor. The peculiarity of TST form *A. vinelandii* [52] is characterized by the presence of only one cysteine residue, which is located in a semicircular loop with sequence stretch -CQTHHR-, which is also the catalytic residue present in the active site. For this reason, damage occurring on the catalytic cysteine is reflected on the enzyme activity that is easy to quantify by means of the Sörbo assay [53]. Therefore, this enzyme can represent a good model for monitoring possible effects on the thiol groups of the proteins due to oxidative damage induced by the formation of radicals.

**Figure 2:**
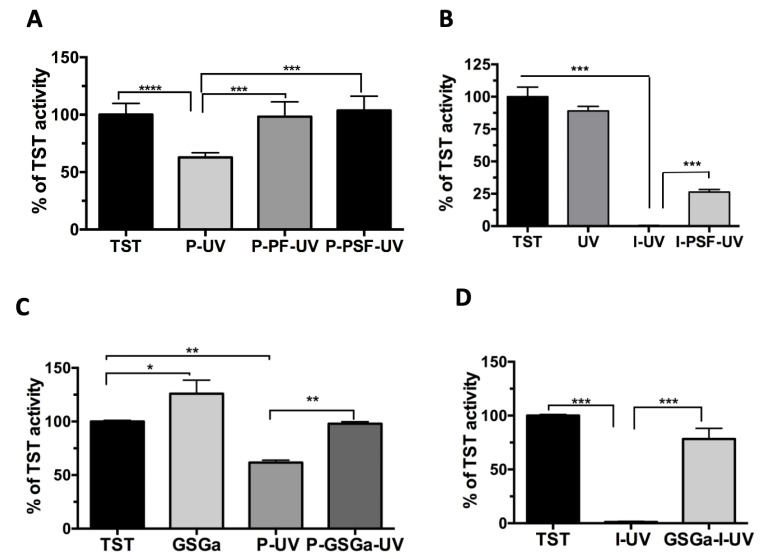
Effects of the photo-polymerization reaction on an enzymatic model. (**A**) Inhibition of 3.9 µM of TST activity in the presence of 10% (*w/v*) of PEGDA 6 kDa (P) and UV exposure for 5 min and protection of the enzymatic activity by the presence of pegylated proteins 10 mg/mL PEG-fibrinogen (PF) and 28.6 µM PEG-Silk Fibroin (PSF) in 50 mM Tris-HCl buffer, pH 8.0; (**B**) TST activity in the presence of 1% (*w/v*) of photo-initiator (I) and under UV for 5 min and in the presence of PSF; (**C**) protection from photo-polymerization damage by addition of 10 µL of GSGa (136 mg (d.w.)/mL) in the solution and (**D**) in the presence of 1% of I (*w/v*). The data were obtained by three or five independent experiments. Error bar indicates S.D. * *p* value ≤ 0.05; ** *p* value ≤ 0.01; *** *p* value ≤ 0.001; **** *p* value ≤ 0.0001.

The TST enzymatic activity following exposure to UVA light at 365 nm either in the presence or in the absence of functional groups that can form radicals is shown in Figure 2. The exposure of the enzyme to UVA in the presence of either PEGDA or Irgacure^®^ 2959 (I) (Figure 2A,B, respectively) resulted in a statistically significant decrease in TST activity that was not observed with the only light exposure The action of the free radicals generated from the Irgacure after UV exposure was particularly harmful, leading to complete inhibition of TST activity (Figure 2B). This inhibition was reduced in both cases by the presence of PEG-proteins in the solution (Figure 2A,B). In fact, the enzymatic activity of TST in the presence of either PEGylated Fibrinogen (PF) or PEGylated Silk Fibroin (PSF), both being precursors to cell-encapsulating hydrogels [20,21], completely protects the TST enzyme from the oxidative damage of UV light, fully preserving its activity (Figure 2A,B), whereas 26.3% of the TST activity was preserved with Irgacure in the presence of PSF (Figure 2B).

These data demonstrate that functionalized proteins which are a part of the hybrid hydrogel scaffold design can provide cell protection from the PhP damage acting as scavengers to free radicals. Having validated the TST assay to quantify oxidative damage associated with PhP, we next sought to measure the protective effects of a glutathione-conjugated garlic extract, named GSGa, which was able to provide a slow-release of H_2_S [30,49]. GSGa was added to the TST solution with PEGDA, and the solution was exposed to UV for 5 min. GSGa was able to protect the TST enzyme from the oxidative damage and/or Micheal-type addiction reaction, as shown in Figure 2C. The GSGa was particularly effective in protecting the TST enzyme in the combined treatment of Irgacure and UV, a condition in which the radical damage to the enzyme completely eliminates its activity (Figure 2D). Despite this protective effect, the preparation of 3D hydrogels using GSGa present during photo-polymerization is not possible due to the radical scavenging property of the organo-sulfur compounds (OSCs) [54] (see Supplementary Materials Figure S2). An alternative approach is to precondition the cells with GSGa or other slow H_2_S-releasing donors, in order to preserve them from the oxidative damage of the hydrogel cross-linking reaction.

### 2.2. Preconditioning of cMSC with a Slow H_2_S-Donor: Effects on Antioxidant Enzyme Expression

**Figure 3:**
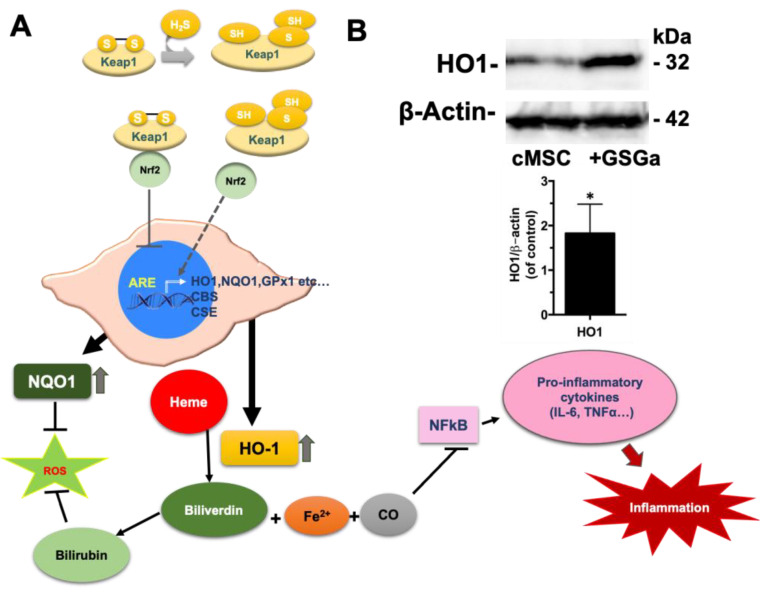
Effects of the GSGa cell preconditioning on the Nrf2-ARE pathway. (**A**) Scheme of the Nrf2-ARE pathway activation by H_2_S. Under basal conditions, Nrf2 binds to its repressor Keap1, which leads to ubiquitination and to the subsequent proteasome degradation. During oxidative stress, free Nrf2 translocates to the nucleus and binds to ARE genes such as HO-1. Upregulated HO-1 catalyzes the degradation of heme into CO, bilirubin, and free iron. CO acts as an inhibitor of the NF-ĸB pathway leading to the decreased expression of pro-inflammatory cytokines, while bilirubin also acts as antioxidant. HO-1 also directly inhibits the proinflammatory cytokines as well as activates the anti-inflammatory cytokines, thus leads to balancing of the inflammatory process [55]; (**B**) Representative western blot analysis of the expression of HO-1 in cMSC cultured for three days in the absence (cMSC) or in the presence (+GSGa) of 680 µg/mL of GSGa. Experiments were performed as three biological replicates; Error bar indicates S.D. * *p* value ≤ 0.05.

Previous studies have shown that GSGa treatment of cardiac cMSC improves their viability, proliferation, and migration rate without affecting their plasticity [49]. Our group also demonstrated that GSGa treatment for three days protects cMSC from induced oxidative stress (by H_2_O_2_ and CoCl_2_ administration), promoting the expression of Antioxidant Responsive Element (ARE) controlled enzymes (Figure 3A), such as NAD(P)H dehydrogenase (quinone 1 reductase) (NQO1), and pro-survival and proliferation, inducing proteins such as Bcl-2 and pERK1/2 [49]. Therefore, we sought to take advantage of these protective effects by preconditioning cMSC with a GSGa treatment prior to their encapsulation in photo-polymerizating hydrogels. In order to first demonstrate the benefits of this, we focused on the ARE promoter activity of the cMSC by measuring the up-regulation of the expression of heme oxygenase-1 (HO-1) after three days of GSGa treatment.

A statistically significant increase in the HO-1 expression was observed, as shown in Figure 3B. NQO1 and HO-1 are enzymes involved in cellular detoxification reactions; in particular, HO-1 is a stress-inducible enzyme that provides protection against oxidative damage with important anti-inflammatory properties [55,56,57,58,59]. Therefore, the GSGa-mediated up-regulation can play an important role in the protection against cellular damage due to the radical polymerization. This effect is related to the H_2_S release that can regulate both NQO1 and HO-1 expression together with other antioxidant enzymes through the effect on the Nrf2-ARE pathway. H_2_S is able to promote the dissociation between Nrf2 and Keap1 by Keap1 sulfhydration at the level of the Cys151 residue [60]. The consequent Nrf2 translocation into the nucleus results in binding to the antioxidant responsive element, inducing downstream antioxidant enzyme expression (Figure 3A), such as NQO1, heme oxygenase (HO-1), glutathione–peroxidase 1 (GPx1), etc. The demonstrated activation of the cellular antioxidant–redox system led us to evaluate the effects of cell preconditioning with GSGa on the resistance to the damage induced by the photo-polymerization reaction.

### 2.3. GSGa as a Preconditioning Agent for Reducing the PhP-Damage on cMSC

**Figure 4:**
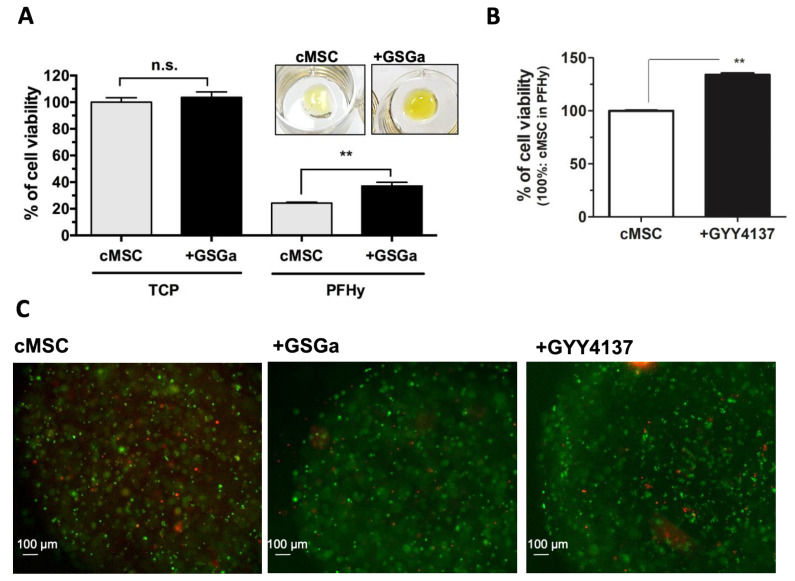
Preconditioning with H_2_S slow-releasing agents protects stem cell 3D culture from photo-polymerization damage. (**A**) WST-1 cell viability assay of cMSC (cMSC) and preconditioned cMSC for three days with 680 µg/mL of GSGa (+GSGa) after PFHy polymerization; 100% was the control represented by cMSC grown on the tissue culture plate (cMSC/TCP); the digital images of the 3D-PHy cell cultures after metabolic WST-1 assay are at the top; (**B**) WST-1 cell viability assay of cMSC embedded in PFHy without (cMSC) and with preconditioning for three days with 100 µM of GYY4137 (+GYY4137); error bar indicates S.D. and ** *p* value ≤ 0.01; (**C**) Representative fluorescence micrographs of LIVE/DEAD assay of cMSC and preconditioned cMSC with GSGa (+GSGa) or with GYY4137 (100 µM) (+GYY4137) that were embedded in PFHy. Scale bars are 100 µm. Experiments were performed as three or four biological replicates. n.s. = not significant.

Given the observed cytoprotective effects of GSGa treatment [49], we evaluated short-term (three days) preconditioning in the context of PhP damage induced during the gelation process. A solution containing cMSC in PFHy precursors with a photo-initiator was exposed to UVA light for 5 min. The cells were cultured in the 3D system for 3 h to let them stabilize before a metabolic assay (WST-1 assay) was carried out to assess their viability. The preconditioned cMSC (three days with 680 µg/mL GSGa) were more resistant to PhPdamage compared to untreated cMSC, showing a relative cell viability of 12.96% ± 2.4 higher than that of the untreated cells (Figure 4A).

**Figure 5:**
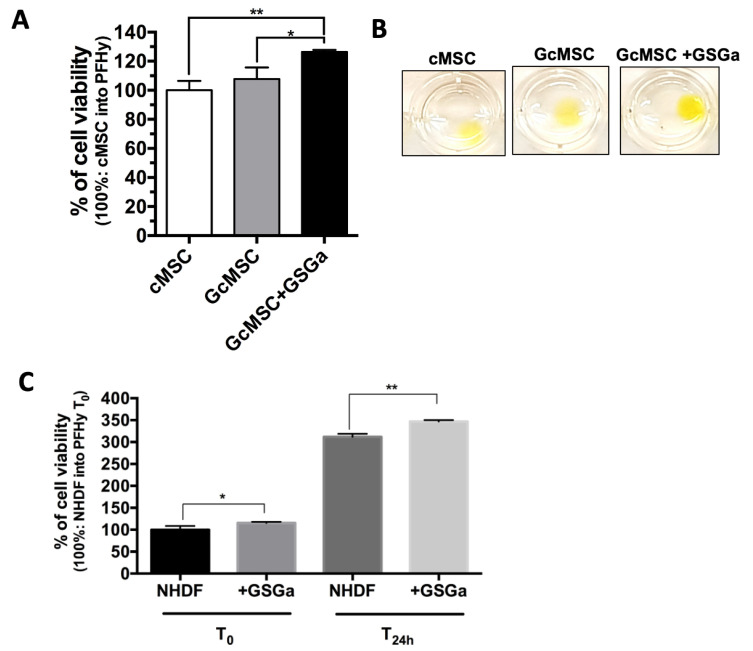
Protection from photo-polymerization damage by GSGa long-term pre-treatment in a 3D cMSC culture system and by GSGa short-term pre-treatment in 3D NHDF culture system. (**A**) WST-1 cell viability assay after PFHy polymerization on stem cells pre-treated for one month with GSGa (140 µg/mL) (GcMSC) and further treated for three days with GSGa (680 µg/mL) (GcMSC+GSGa) before embedding; 100% viability is represented by untreated cMSC embedded in the hydrogel; (**B**) digital images of the hydrogels after WST-1 assay; (**C**) cell viability assay of NHDF and preconditioned NHDF for three days with GSGa (680 µg/mL) (+GSGa) before embedding in PFHy for 3 h (T_0_) and after 24 h (T_24h_). Each result was obtained from three independent experiments. Error bar indicates S.D. * *p* value ≤ 0.05; ** *p* value ≤ 0.01.

The preconditioning of cMSC using the synthetic H_2_S-donor GYY4137 (100 µM) also resulted in an improved viability following PhP damage, as shown in Figure 4B; the cell viability increased by about 30% compared to the untreated cells in the PFHy. This improvement in the relative cell viability was also evident in the fluorescence micrographs of the cMSC in PFHy following live/dead assay (Figure 4C). Long-term preconditioning has also been proposed as a means of increasing the cytoprotective effects of the H_2_ -donor treatment. The long-term treatment, referred to as GcMSC, involves the preconditioning of cMSC for 30 days in the presence of 140 µg/mL GSGa [49]. GcMSC in the PFHy were found to be more protected from the PhP damage compared to untreated cMSC, particularly after additional treatment for three days with GSGa (680 µg/mL) before embedding in the PFHy (Figure 5A,B). With this pre-treatment, an increase of 26.4% ± 1.4 of the relative cell viability was observed, which was 18.69% ± 4.8 higher than the observed increase with GcMSC. The results suggest that a combined approach could be the optimal solution for increasing the cell survival and stability in the 3D-PFHy systems. The cell survival after photo-polymerization due to the GSGa preconditioning was also assessed on another cell line. Preconditioned NHDF (three days with 680 µg/mL GSGa) showed a significant increase in the cell viability of 15.48% ± 5.2 and 35.1% ± 4.4 compared to the untreated controls when assessed either after 3 h (T_0_) or 24 h (T_24h_) from the PFHy photo-polymerization, respectively (Figure 5C). These findings are in agreement with the above demonstrated activation of the Nrf2-ARE pathway by GSGa pretreatment and, in general, with the antioxidant properties of the H_2_S-releasing agents, showing a simple method to obtain a reduction in cellular oxidative stress without using antioxidant compounds that could inhibit the radical-based polymerization process.

### 2.4. GSGa Treatment Improves cMSC Proliferation in 3D-Hydrogel Microspheres

**Figure 6:**
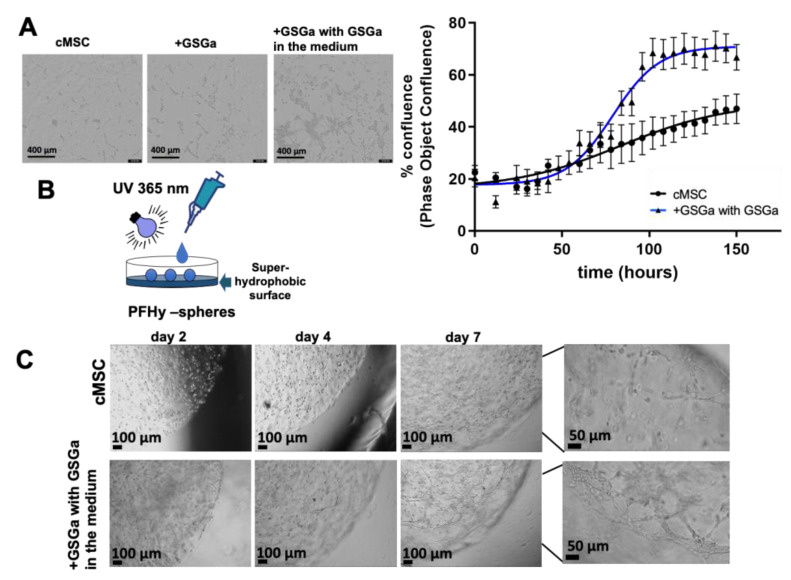
Effect of GSGa in culture medium on the growth and proliferation of cMSC in 3D-PFHy systems. (**A**) Bright-field micrographs of cells untreated (cMSC) in the PFHy, pre-treated for three days with GSGa (680 µg/mL) before embedding in PFHy (+GSGa), and pre-treated cells with 680 µg/mL of GSGa added to the medium, obtained by IncuCyte after one day of cell growth. Scale bars are 400 µm. The % of confluence after six days of growth for both cMSC and +GSGa with GSGa in the medium was obtained using phase object confluence analysis by Incucyte; (**B**) Schematic representation of the PFHy sphere production on a super-hydrophobic surface; (**C**) Bright-field micrographs of untreated cMSC and pre-treated with GSGa for three days before embedding with or without GSGa in the culture medium. Images after two, four, and seven days of growth are reported. Scale bars are 100 µm and 50 µm.

The effect of the GSGa preconditioning on the morphology, growth, and proliferation of cMSC in the PFHy scaffolds was also evaluated for longer 3D culture durations. The GSGa preconditioning (+GSGa) increased the number of intercellular connections and appeared to enhance cell proliferation and cell–cell interaction based on phase-contrast micrographs of the constructs after one week (Figure 6A). Quantitative cell proliferation was monitored in these constructs continuously for up to one week using the IncuCyte^®^ S3 Live-Cell system. The quantitative results confirm the micrograph observations that preconditioned cells (+GSGa) grown in PFHy with culture medium containing GSGa are more prolific than the untreated controls.

Microspheric carriers were used to further evaluate these differences. The microcarriers were based on PFHy for 3D cell-growth, where cMSC were encapsulated into the hydrogel microspheres (5 to 20 µL) produced by dripping the precursor solution onto a super-hydrophobic surface, as described in Figure 6B. The addition of GSGa (680 µg/mL) in the culture medium increased the formation of lamellipodia and intercellular connections in the 3D-hydrogel microspheres, as shown in Figure 6C, suggesting once again that a combined approach could be more effective. In agreement with the data reported with the larger constructs, preconditioned cMSC (+GSGa) cultured in the microspheres for one week formed more lamellipodia and intercellular connections in the presence of 680 µg/mL GSGa in the medium when compared to controls (Figure 6C and video in Supplementary Materials).

**Figure 7:**
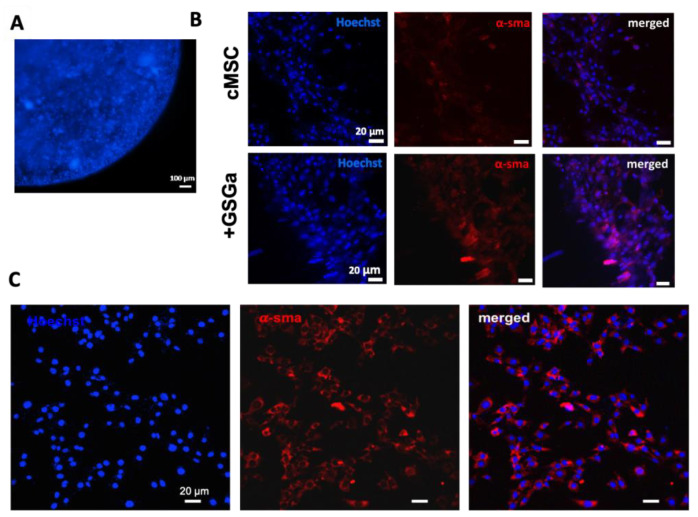
Effects of GSGa treatment of cMSC in 3D-PFHy culture systems on α-SMA. (**A**) Fluorescence micrograph of cMSC embedded in the PFHy microsphere (5 µL); after one week of growth, the nuclei were stained with Hoechst, using a protocol for staining of live cells. Scale bar is 100 µm; (**B**) cMSC embedded in PFHy (cMSC) and treated with GSGa (680 µg/mL) added to the culture medium (+GSGa) after seven days of growth. Z-stacks were obtained from the overlapping of 21 slices. Scale bars are 20 µm; (**C**) cMSC embedded in PFHy after 10 days of culture in complete medium containing 680 μg/mL GSGa. Nuclei were in vivo stained with Hoechst dye, and α-SMA is shown in red. Scale bars represent 20 μm.

Beyond the apparent improvements to cell proliferation associated with the GSGa treatment, we assessed improvements in phenotypic expression associated with cMSC. Toward these aims, preliminary immunofluorescence characterization of the cells was performed after one week of 3D culture in the microspheres (Figure 7). The confocal immunofluorescence micrographs of cells grown into 3D-PFHy scaffolds in the presence and in the absence of GSGa (680 µg/mL) are shown in Figure 7B. Hoechst staining for live cell detection was performed (Figure 7A), and after fixation, the cells were stained with anti-α-smooth muscle actin (α-SMA) antibodies. α-SMA is one of the more prevalent muscle specific biomolecular markers. The results indicated that many nuclei were visible within the microsphere, and their integrity suggested good cell viability with limited pyknosis.

An increased expression of α-SMA was also observed in the GSGa-treated samples (Figure 7B). The stability of the 3D culture and the α-SMA protein expression were further monitored with immunofluorescence analysis after 10 days of growth (Figure 7C). These results indicate that the beneficial effects of the GSGa treatment were preserved in the 3D culture conditions. Notably, these data are in agreement with previously reported results by western blot and 2D culture immunofluorescence analyses [49] and show that the GSGa treatment protects cMSC from PhP damage and also improves their proliferation in the PFHy scaffold.

Thanks to the cytoprotective properties of H_2_S-donors, the preconditioning of stem cells before their transplantation has recently gained attention [48,49,61]. However, not many studies have investigated the use of H_2_S-donors in tissue engineering, and most of them are focused on a short-term (h) preconditioning of stem cells prior to the transplantation. Our results suggest that cells preconditioned for a few days with slow H_2_S-releasing agents might represent a promising approach for the future preservation of stem cells before embedding in the 3D-hydrogel scaffolds obtainable by UV-photo-polymerization.

## 3. Materials and Methods

### 3.1. Recombinant TST Production

Recombinant thiosulfate: cyanide sulfurtransferase (TST, rhodanese, RhdA, EC. 2.8.1.1) from *Azotobacter vinelandii* was produced and purified as previously described [51,52]. Briefly, the plasmid pQER1 containing the gene coding for TST with a N-terminal His-tag was used to transform the *Escherichia coli* strain, and overexpression of the recombinant protein was induced by the addition of isopropyl thio-β-D-galactoside to a mid-exponential culture. TST was purified by chromatography on a Ni-NTA column. The His-tagged protein was eluted by the addition of 200 mM imidazole and precipitation in 75% saturated ammonium sulfate. The protein concentration was determined using ε^0.1%^_280_ = 1.3, and the molecular weight of 31 kDa was estimated by SDS/PAGE.

### 3.2. TST Activity

TST activity was tested using the Sörbo assay [53]–where thiocyanate production from thiosulfate and cyanide is assessed–obtaining an enzymatic activity of 64.06 U/mg. Briefly, the Sörbo assay was performed as follows: the recombinant TST enzyme was incubated at 37 °C in a reaction mixture (650 μL of 58 mM KCN and 58 mM sodium thiosulfate in 50 mM Tris-HCl buffer, pH 8.0). The reaction was stopped after 1 min by adding 100 μL of 15% formaldehyde and 250 µL Sörbo reagent (100 g of ferric nitrate and 200 mL of 65% nitric acid per 1500 mL), developing colour. The product was monitored by reading the absorbance at 460 nm.

In order to evaluate the photo-polymerization damage caused to TST activity, the Sörbo assay was performed in a 3.9 μM of TST solution in the presence and in the absence of 10% (*w/v*) PEGDA 6 kDa, 365 nm UV exposure, 1% of Irgacure^®^2959 (I)–at the concentration used for hydrogel synthesis–after 5 min of incubation at room temperature. The PEGylated Fibrinogen (PF) and PEGylated Silk Fibroin (PSF) solutions used for the analysis of the PhP damage caused to TST activity were prepared as previously described [20,21].

### 3.3. Garlic Water-Soluble Extract Production from Allium sativum L.

The garlic water-soluble extract was produced as previously described [30,49]. Briefly, 5 g of garlic cloves was crushed in liquid N_2_ for about 10 min in the presence of 50 mM Tris-HCl buffer, pH 7.5, with 100 mM reduced glutathione (GSH) (Sigma-Aldrich, Milan, Italy). After centrifugation, the water-soluble fraction was stored at −20 °C for molecular characterization with RP-HPLC. We performed RP-HPLC analysis using mod. LC-10AVP (Shimadzu, Milan, Italy), equipped with a UV detector (Shimadzu, Milan, Italy) and a C18 column (150 mm × 4.6 mm, 5 µm, CPS Analitica, Rome, Italy), using 0.1% trifluoracetic acid as solvent A and 80% CH_3_CN, 0.1% trifluoracetic acid as solvent B and with a solvent B gradient (0–5 min, 0%; 5–55 min, 60%; 55–60 min, 60% and 65–85 min 90%). The elute was monitored at 220 nm. To determine the dry weight of GSGa and its concentration, 100 µL of the extract was lyophilized.

### 3.4. MB-Assay for H_2_S Release

H_2_S production by GSGa and GYY4137 was evaluated by methylene blue assay (see supplementary material Figure S1) as described previously [30]. A solution of 150 µL containing 25 µL of the extract, 50 mM Tris HCl, pH 7.4, and 1 mM dithiothreitol (DTT) (Sigma-Aldrich, Milan, Italy) was incubated at 37 °C on a shaker for 30 min. After incubation, 20 µL of a solution I (20 mM *N′,N′*-dimethyl-p-phenylene-diamine-dihydrochloride in 7.2 M HCl) and 20 µL of a solution II (30 mM FeCl_3_ in 1.2 M HCl) were added and after 10 min of mixing at room temperature, the absorbance at a wavelength of 670 nm was measured. A standard curve was obtained using Na_2_S (see Figure S1 in Supplementary Materials).

### 3.5. Western Blotting Analysis

Protein extraction from cMSC was performed using RIPA buffer (100 µL) containing a protease inhibitor cocktail (Sigma–Aldrich, Milan, Italy) and pervanadate (Sigma–Aldrich, Italy) as a phosphatase inhibitor. After incubation for 90 min in ice, lysates were sonicated at 0 °C for 10 s and then centrifuged for 10 min at 8000 rpm at 4 °C. BCA protein assay (Sigma–Aldrich, Milan, Italy) was used to determine the protein content, and the SDS-PAGE of cell extracts (30 µg of protein) was performed using 15% polyacrylamide gel. For electro-blotting, PVDF membranes (Sigma–Aldrich, Italy) were used and were then blocked and probed with primary monoclonal antibody (Ab-HO-1) (Sigma–Aldrich, Italy) overnight at 4 °C. After that, immunoblots were processed using a secondary antibody (dilution 1:3000) (Sigma–Aldrich, Milan, Italy) for 4 h at room temperature. Immunoblot with Ab-β-actin mouse (Sigma–Aldrich, Milan, Italy) was also probed to control the protein loading. Immunoblots were probed with a Super Signal West Pico kit (Thermo Scientific, Milan, Italy) to visualize signal, followed by exposure to a Fluorchem Imaging system (Alpha Innotech Corporation-Analitica De Mori, Milan, Italy).

### 3.6. Hydrogel Scaffold Preparation

The PFHy precursor solution was obtained according to a published protocol [20]. Briefly, the PFHy precursor solution was solubilized in PBS, pH 7.4, at a final concentration of 8 mg/mL, and to favour the gelation process, 0.5% *v/v* PEGDA (10 kDa) of a 30% PEGDA solution was also added. The assembly of PFHy was achieved via free-radical polymerization by adding 1% *v/v* of a photo-initiator stock solution containing 10% *w/v* Irgacure^®^ 2959 (Ciba Specialty Chemicals, Basel, Switzerland) in 70 vol% ethanol; thus, the final concentration of Irgacure in the precursor solution was 0.1% *w/v*, by exposing the sample to UV light (365 nm, 4–5 mW cm^–2^) for 5 min. The material underwent a phase change from a sol to a gel. For microsphere production, the precursor solution was dropped on a super-hydrophobic surface obtained by deposing on a microscope glass slide a solution of AEROSIL^TM^ 1% *w/v* in acetone.

### 3.7. 3D Cell Cultures and Cell Viability Assay

Sca-1^+^ Lin^−^ cMSC were isolated from auricular biopsies taken during coronary artery bypass surgery, as described elsewhere [62]. cMSC and NHDF were cultured in Dulbecco’s modified Eagle medium (DMEM) (Gibco, Thermo Fisher Scientific, Milan, Italy) supplemented with 10% of fetal bovine serum (FBS) (*v*/*v*) (Gibco, Thermo Fisher Scientific, Milan, Italy), 2 mM l-Glutamine, 100 U/mL penicillin, and 100 µg/mL streptomycin (hereafter referred to as “complete medium”) at 37 °C and with 5% CO_2_. The cell preconditioning was performed culturing the cMSC or NHDF for three days in the cell culture medium with 680 µg (d.w.)/mL of GSGa. The GSGa was added in the medium only after cell adhesion to the well. After detachment by treatment with trypsin- EDTA, cells were resuspended in 60 μL of a hydrogel precursor solution at a cell density of 1 × 10^6^ cells/mL. The solution was placed into a sterilized teflon mold (5-mm inner diameter), and the photo-polymerization was performed according to the protocol described above. The cell-seeded hydrogels were cultivated in complete medium. The cell viability was quantified by WST- 1 metabolic colorimetric assay [63]. Briefly, the WST-1 assay (4-[3-(4-lodophenyl)-2-(4-nitrophenyl)-2H-5-tetrazolio]-1,3-benzene disulfonate) (Roche Diagnostics, Sigma-Aldrich, Milan, Italy) was performed by incubating the hydrogel samples for 3 h in complete DMEM (without phenol-red) in the presence of 5% (*v/v*) cell proliferation reagent WST-1 at 37 °C and in 5% CO_2_. The absorbance of the medium was evaluated using an iMarkTM Microplate Reader (Bio-Rad) at a 450 nm wavelength. The viability of MSC (1 × 10^6^ cells/mL) with respect to PFHy was also monitored by fluorescence microscopy using a LIVE/DEAD^®^ Cell Imaging Kit (488/570) (Molecular Probes, Life Technologies, Thermo Fisher Scientific, Milan, Italy). The IncuCyte^®^ S3 Live-Cell system was used for monitoring the cell proliferation in the 3D-PFHy. Growth of cMSC (1 × 10^6^ cells/mL) embedded in a PFHy monolayer was monitored for one week, acquiring bright-field images (16 images per well) every six h with 10× magnification.

### 3.8. Immunofluorescence Microscopy

Immunofluorescence of 3D-PFHy cMSC cultures was performed by in-vivo nuclei staining with Hoechst 33,342 (Sigma–Aldrich, Italy); then, the gels were washed in PBS, fixed in 4% paraformaldehyde (PFA) in PBS for 30 min at room temperature, permeabilized with 0.3% Triton X-100 for 5 min, and maintained in a blocking buffer (10% *v/v* FBS, 0.1% *v/v* Triton X-100, and 1% *w/v* glycine in PBS) overnight at 4 °C. Hydrogels were then incubated overnight at 4 °C with 1:200 *v/v* Ab-α-smooth muscle actin (α-SMA) in PBS with 1% albumin with 20 mM Gly solution, followed by 3 h of incubation with the appropriate 1:200 *v/v* Alexa fluorochrome-conjugated secondary antibodies in 20 mM Gly-PBS at room temperature. Confocal microscopy was performed using a Nikon Eclipse Ti.

### 3.9. Statistical Analysis

The statistical analysis was performed using GraphPad Prism version 6.0 for Windows (GraphPad Software, San Diego, CA, USA). Data from three or five independent experiments were quantified and analyzed for each variable using a one-tailed Student’s *t*-test or one-way ANOVA. A *p* value of <0.05 was considered to be statistically significant. Standard deviation or the standard error mean was calculated and reported for each type of sample.

## 4. Conclusions

The detrimental effects associated with free-radical polymerization can be a major drawback to the use of photo-polymerization in the preparation of hydrogel-based 3D scaffolds for cell therapy and bioprinting. We demonstrated that PhP damage, due to either radical oxidation or Michael-addition reactions at the level of cellular proteins (i.e., cysteine residues), can be significantly reduced by the presence of protein-based hydrogel precursor constituents or slow H_2_S-releasing donors in the pre-gel solution. More importantly, we sought to mitigate the PhP damage caused by free-radicals during the cross-linking reaction using a simple yet effective preconditioning of the cells with an H_2_S-donor, just prior to their encapsulation in a PFHy scaffold. PhP damage was significantly reduced by the activation of the antioxidant cellular system using cell preconditioning with GSGa, a phytochemical H_2_S-donor. The activation of the Nrf2-ARE pathway, which has a relevant action on the anti-oxidative response of the cell, by GSGa preconditioning was demonstrated. The activation of the expression of both enzymes NQO1 [49] and HO-1 of the Nrf2-ARE pathway can induce a cytoprotective effect reducing the PhP damage. Recent studies have demonstrated that HO-1 and its products, such as biliverdin, could be protective agents during transplantation by reduction of ischemia–reperfusion injury [64,65,66]. Moreover, an increase in the survival and the function of transplanted islets due to HO-1 induction was also observed during the transplantation of pancreatic islets for treatment of type 1 diabetes [67,68]. Therefore, the demonstrated cytoprotectant, anti-inflammatory, and immunomodulator role of HO-1 [55] (see Figure 3A) could play a relevant role both in the regenerative tissue implantation and in stem cell carrier therapy, promoting a reduction of the local inflammatory process.

H_2_S donors, such as GSGa, are promising exploitable tools to overcome the massive cell death that occurs after stem cell implantation in the site of injury for stem cell-based therapy. Previously, we also demonstrated that cell preconditioning does not affect the cell plasticity [49]. Therefore, the here demonstrated effectiveness in preventing PhP damage of the phytochemical H_2_S donor paves the way for the use of slow H_2_S-releasing agents in pre-treatment of cell cultures for a wide range of tissue engineering applications, including the pioneering 3D-bioprinting technologies and tissue repair based on stem cell-delivery systems.

## Supplementary Materials

The following are available online at https://www.mdpi.com/article/10.3390/ijms22116095/s1.

## Abbreviations

α-SMA	α-smooth muscle actin
ARE	Antioxidant response element
BCA	bicinchoninic acid
BM-MSC	bone marrow mesenchymal stem cells
cMSC	Sca-1^+^ Lin^−^ human cardiac mesenchymal stem cells
DMEM	Dulbecco’s modified Eagle medium
FBS	Fetal Bovine Serum
H_2_S	hydrogen sulfide
HO-1	heme oxygenase 1
I	photo-initiator
Keep1	Kelch ECH associating protein 1
NHDF	normal human dermal fibroblasts
NQO1	NAD(P)H quinoneoxidoreductase 1
Nrf2	nuclear factor erythroid 2-related factor 2
OSCs	Organo-sulfur compounds
SCs	stem cells
PhP damage	photo-polymerization damage/photo-polymerization induced damage
TST	thiosulfate: cyanide sulfurtransferase enzyme

## References

[1] Satija N., Singh V.K., Verma Y.K., Gupta P., Sharma S., Afrin F., Sharma M., Sharma P., Tripathi R.P., Gurudutta G.U. (2009). Mesenchymal stem cell-based therapy: A new paradigm in regenerative medicine. J. Cell. Mol. Med..

[2] Burdick J.A., Mauck R., Gerecht S. (2016). To Serve and Protect: Hydrogels to Improve Stem Cell-Based Therapies. Cell Stem Cell.

[3] Sanina C., Hare J.M. (2015). Mesenchymal Stem Cells as a Biological Drug for Heart Disease: Where Are We With Cardiac Cell-Based Therapy?. Circ. Res..

[4] Mazini L., Rochette L., Amine M., Malka G. (2019). Regenerative Capacity of Adipose Derived Stem Cells (ADSCs), Comparison with Mesenchymal Stem Cells (MSCs). Int. J. Mol. Sci..

[5] Copland I.B. (2011). Mesenchymal stromal cells for cardiovascular disease. J. Cardiovasc. Dis. Res..

[6] Song H., Cha M.-J., Song B.-W., Kim I.-K., Chang W., Lim S., Choi E.J., Ham O., Lee S.-Y., Chung N. (2010). Reactive Oxygen Species Inhibit Adhesion of Mesenchymal Stem Cells Implanted into Ischemic Myocardium via Interference of Focal Adhesion Complex. Stem Cells.

[7] Abdelwahid E., Kalvelyte A., Stulpinas A., De Carvalho K.A.T., Guarita-Souza L.C., Foldes G. (2016). Stem cell death and survival in heart regeneration and repair. Apoptosis.

[8] Amer M.H., Rose F.R.A.J., Shakesheff K.M., White L.J. (2018). A biomaterials approach to influence stem cell fate in injectable cell-based therapies. Stem Cell Res. Ther..

[9] Baldari S., Di Rocco G., Piccoli M., Pozzobon M., Muraca M., Toietta G. (2017). Challenges and Strategies for Improving the Re-generative Effects of Mesenchymal Stromal Cell-Based Therapies. Int. J. Mol. Sci..

[10] Qian L., Shim W., Gu Y., Shirhan M., Lim K.P., Tan L.P., Lim C.H., Sin Y.K., Wong P. (2012). Hemodynamic Contribution of Stem Cell Scaffolding in Acute Injured Myocardium. Tissue Eng. Part A.

[11] Seliktar D. (2012). Designing Cell-Compatible Hydrogels for Biomedical Applications. Science.

[12] Kopeček J. (2007). Hydrogel biomaterials: A smart future?. Biomaterials.

[13] Bryant S.J., Vernerey F.J. (2017). Programmable Hydrogels for Cell Encapsulation and Neo-Tissue Growth to Enable Personalized Tissue Engineering. Adv. Health Mater..

[14] Tan H., Marra K.G. (2010). Injectable, Biodegradable Hydrogels for Tissue Engineering Applications. Materials.

[15] Vunjak-Novakovic G., Lui K.O., Tandon N., Chien K.R. (2011). Bioengineering heart muscle: A paradigm for regenerative med-icine. Ann. Rev. Biomed. Eng.

[16] Gasperini L., Mano J.F., Reis R.L. (2014). Natural polymers for the microencapsulation of cells. J. R. Soc. Interface.

[17] Elisseeff J., Anseth K., Sims D., McIntosh W., Randolph M., Langer R. (1999). Transdermal photopolymerization for minimally invasive implantation. Proc. Natl. Acad. Sci. USA.

[18] Ferreira P., Coelho J., Gil M.H. (2008). Development of a new photocrosslinkable biodegradable bioadhesive. Int. J. Pharm..

[19] West J.L., Hubbell J.A. (1999). Polymeric Biomaterials with Degradation Sites for Proteases Involved in Cell Migration. Macromolecules.

[20] Almany L., Seliktar D. (2005). Biosynthetic hydrogel scaffolds made from fibrinogen and polyethylene glycol for 3D cell cultures. Biomaterials.

[21] Ciocci M., Cacciotti I., Seliktar D., Melino S. (2018). Injectable silk fibroin hydrogels functionalized with microspheres as adult stem cells-carrier systems. Int. J. Biol. Macromol..

[22] Fedorovich N.E., Oudshoorn M.H., van Geemen D., Hennink W.E., Alblas J., Dhert W.J. (2009). The effect of photopolymeri-zation on stem cells embedded in hydrogels. Biomaterials.

[23] Gopinathan J., Noh I. (2018). Click Chemistry-Based Injectable Hydrogels and Bioprinting Inks for Tissue Engineering Applications. Tissue Eng. Regen. Med..

[24] Brown T.E., Carberry B.J., Worrell B.T., Dudaryeva O.Y., McBride M.K., Bowman C.N., Anseth K.S. (2018). Photopolymerized dynamic hydrogels with tunable viscoelastic properties through thioester exchange. Biomaterials.

[25] Nulty J., Freeman F.E., Browe D.C., Burdis R., Ahern D.P., Pitacco P., Bin Lee Y., Alsberg E., Kelly D.J. (2021). 3D bioprinting of prevascularised implants for the repair of critically-sized bone defects. Acta Biomater..

[26] Costantini M., Testa S., Fornetti E., Fuoco C., Riera C.S., Nie M., Bernardini S., Rainer A., Baldi J., Zoccali C. (2021). Biofabricating murine and human myo-substitutes for rapid volumetric muscle loss restoration. EMBO Mol. Med..

[27] Bryant S.J., Nuttelman C.R., Anseth K.S. (2000). Cytocompatibility of UV and visible light photoinitiating systems on cultured NIH/3T3 fibroblasts in vitro. J. Biomater. Sci. Polym. Ed..

[28] Sabnis A., Rahimi M., Chapman C., Nguyen K.T. (2009). Cytocompatibility studies of an in situ photopolymerized ther-moresponsive hydrogel nanoparticle system using human aortic smooth muscle cells. J. Biomed. Mater. Res. A.

[29] Cao Q., Zhang L., Yang G., Xu C., Wang R. (2010). Butyrate-stimulated H2S Production in Colon Cancer Cells. Antioxid. Redox Signal..

[30] Bhuiyan A.I., Papajani V.T., Paci M., Melino S.M. (2015). Glutathione-Garlic Sulfur Conjugates: Slow Hydrogen Sulfide Releasing Agents for Therapeutic Applications. Molecules.

[31] Bolton S., Cerda M., Gilbert A.K., Pluth M.D. (2019). Effects of sulfane sulfur content in benzyl polysulfides on thiol-triggered H2S release and cell proliferation. Free Radic Biol. Med..

[32] Rose P., Moore P.K., Ming S.H., Nam O.C., Armstrong J.S., Whiteman M. (2005). Hydrogen sulfide protects colon cancer cells from chemopreventative agent beta-phenylethyl isothiocyanate induced apoptosis. World J. Gastroenterol..

[33] Cai W., Wang M., Ju L., Wang C., Zhu Y. (2010). Hydrogen sulfide induces human colon cancer cell proliferation: Role of Akt, ERK and p21. Cell Biol. Int..

[34] Chuah S.C., Moore P.K., Zhu Y.Z. (2007). S-allylcysteine mediates cardioprotection in an acute myocardial infarction rat model via a hydrogen sulfide-mediated pathway. Am. J. Physiol. Circ. Physiol..

[35] Deplancke B., Gaskins H.R. (2003). Hydrogen sulfide induces serum-independent cell cycle entry in nontransformed rat intestinal epithelial cells. FASEB J..

[36] Hu L.-F., Lu M., Wu Z.-Y., Wong P.T.-H., Bian J.-S. (2008). Hydrogen Sulfide Inhibits Rotenone-Induced Apoptosis via Preservation of Mitochondrial Function. Mol. Pharmacol..

[37] Wang H., Pao J., Lin S., Sheen L. (2012). Molecular mechanisms of garlic-derived allyl sulfides in the inhibition of skin cancer progression. Ann. N. Y. Acad. Sci..

[38] Li M., Min J.-M., Cui J.-R., Zhang L.-H., Wang K., Valette A., Davrinche C., Wright M., Leung-Tack J. (2002). Z-Ajoene Induces Apoptosis of HL-60 Cells: Involvement of Bcl-2 Cleavage. Nutr. Cancer.

[39] Yin X., Zhang R., Feng C., Zhang J., Liu D., Xu K., Wang X., Zhang S., Li Z., Liu X. (2014). Diallyl disulfide induces G2/M arrest and promotes apoptosis through the p53/p21 and MEK-ERK pathways in human esophageal squamous cell carcinoma. Oncol Rep..

[40] Xiao D., Herman-Antosiewicz A., Antosiewicz J., Xiao H., Brisson M., Lazo J.S., Singh S.V. (2005). Diallyl trisulfide-induced G2–M phase cell cycle arrest in human prostate cancer cells is caused by reactive oxygen species-dependent destruction and hyperphosphorylation of Cdc25C. Oncogene.

[41] Xiao D., Zeng Y., Hahm E.-R., Kim Y.-A., Ramalingam S., Singh S.V. (2009). Diallyl trisulfide selectively causes Bax- and Bak-mediated apoptosis in human lung cancer cells. Environ. Mol. Mutagen..

[42] Murai M., Inoue T., Suzuki-Karasaki M., Ochiai T., Ra C., Nishida S., Suzuki-Karasaki Y. (2012). Diallyl trisulfide sensitizes human melanoma cells to TRAIL-induced cell death by promoting endoplasmic reticulum-mediated apoptosis. Int. J. Oncol..

[43] Chandra-Kuntal K., Lee J., Singh S.V. (2013). Critical role for reactive oxygen species in apoptosis induction and cell migration inhibition by diallyl trisulfide, a cancer chemopreventive component of garlic. Breast Cancer Res. Treat..

[44] Dirsch V.M., Gerbes A.L., Vollmar A.M. (1998). Ajoene, a compound of garlic, induces apoptosis in human promyeloleukemic cells, accompanied by generation of reactive oxygen species and activation of nuclear factor kappaB. Mol. Pharmacol..

[45] Osipov R.M., Robich M.P., Chan V., Clements R.T., Deyo R.J., Feng J., Szabo C., Sellke F.W. (2010). Effect of hydrogen sulfide on myocardial protection in the setting of cardioplegia and cardiopulmonary bypass?. Interact. Cardiovasc. Thorac. Surg..

[46] Yang R., Liu Y., Shi S. (2016). Hydrogen Sulfide Regulates Homeostasis of Mesenchymal Stem Cells and Regulatory T Cells. J. Dent. Res..

[47] Xie X., Sun A., Zhu W., Huang Z., Hu X., Jia J., Zou Y., Ge J. (2012). Transplantation of Mesenchymal Stem Cells Preconditioned with Hydrogen Sulfide Enhances Repair of Myocardial Infarction in Rats. Tohoku J. Exp. Med..

[48] Zhang Q., Liu S., Li T., Yuan L., Liu H., Wang X., Wang F., Wang S., Hao A., Liu D. (2016). Preconditioning of bone marrow mesenchymal stem cells with hydrogen sulfide improves their therapeutic potential. Oncotarget.

[49] Di Giovanni E., Buonvino S., Amelio I., Melino S. (2020). Glutathione-Allylsulfur Conjugates as Mesenchymal Stem Cells Stim-ulating Agents for Potential Applications in Tissue Repair. Int. J. Mol. Sci..

[50] Seliktar D., Dikovsky D., Napadensky E. (2013). Bioprinting and Tissue Engineering: Recent Advances and Future Perspectives. Isr. J. Chem..

[51] Colnaghi R., Pagani S., Kennedy C., Drummond M. (1996). Cloning, Sequence Analysis and Overexpression of the Rhodanese Gene of Azotobacter vinelandii. JBIC J. Biol. Inorg. Chem..

[52] Sabelli R., Iorio E., De Martino A., Podo F., Ricci A., Viticchiè G., Rotilio G., Paci M., Melino S.M. (2008). Rhodanese-thioredoxin system and allyl sulfur compounds. FEBS J..

[53] Sörbo B. (1957). A colorimetric method for the determination of thiosulfate. Biochim. Biophys. Acta Bioenerg..

[54] Ciocci M., Iorio E., Carotenuto F., Khashoggi H.A., Nanni F., Melino S. (2016). H2S-releasing nanoemulsions: A new formulation to inhibit tumor cells proliferation and improve tissue repair. Oncotarget.

[55] Ahmed S.M.U., Luo L., Namani A., Wang X.J., Tang X. (2017). Nrf2 signaling pathway: Pivotal roles in inflammation. Biochim. Biophys. Acta Mol. Basis Dis..

[56] Otterbein L., Sylvester S.L., Choi A.M. (1995). Hemoglobin provides protection against lethal endotoxemia in rats: The role of heme oxygenase-1. Am. J. Respir. Cell Mol. Biol..

[57] Otterbein L.E., Kolls J.K., Mantell L.L., Cook J.L., Alam J., Choi A.M. (1999). Exogenous administration of heme oxygenase-1 by gene transfer provides protection against hyperoxia-induced lung injury. J. Clin. Investig..

[58] Otterbein L.E., Bach F.H., Alam J., Soares M.P., Lu H.T., Wysk M.A., Davis R.J., Flavell R.A., Choi A.M.K. (2000). Carbon monoxide has anti-inflammatory effects involving the mitogen-activated protein kinase pathway. Nat. Med..

[59] Lee T.-S., Chau L.-Y. (2002). Heme oxygenase-1 mediates the anti-inflammatory effect of interleukin-10 in mice. Nat. Med..

[60] Altaany Z., Yang G., Wang R. (2013). Crosstalk between hydrogen sulfide and nitric oxide in endothelial cells. J. Cell. Mol. Med..

[61] Sart S., Ma T., Li Y. (2014). Preconditioning Stem Cells for In Vivo Delivery. BioRes. Open Access.

[62] Forte A., Rinaldi B., Sodano L., Berrino L., Rossi F., Finicelli M., Grossi M., Cobellis G., Botti C., De Feo M. (2011). Stem Cell Therapy for Arterial Restenosis: Potential Parameters Contributing to the Success of Bone Marrow-Derived Mesenchymal Stromal Cells. Cardiovasc. Drugs Ther..

[63] Koyanagi M., Kawakabe S., Arimura Y. (2015). A comparative study of colorimetric cell proliferation assays in immune cells. Cytotechnology.

[64] Tang L.-M., Wang Y.-P., Wang K., Pu L.-Y., Zhang F., Li X.-C., Kong L.-B., Sun B.-C., Li G.-Q., Wang X.-H. (2007). Exogenous Biliverdin Ameliorates Ischemia-Reperfusion Injury in Small-for-Size Rat Liver Grafts. Transplant. Proc..

[65] Sugimoto R., Tanaka Y., Noda K., Kawamura T., Toyoda Y., Billiar T.R., McCurry K.R., Nakao A. (2012). Preservation solution supplemented with biliverdin prevents lung cold ischaemia/reperfusion injury. Eur. J. Cardio-Thorac. Surg..

[66] Yamashita K., McDaid J., Öllinger R., Tsui T., Berberat P.O., Usheva A., Csizmadia E., Smith R.N., Soares M.P., Bach F.H. (2004). Biliverdin, a natural product of heme catabolism, induces tolerance to cardiac allografts. FASEB J..

[67] Pileggi A., Molano R.D., Berney T., Cattan P., Vizzardelli C., Oliver R., Fraker C., Ricordi C., Pastori R.L., Bach F.H. (2001). Heme Oxygenase-1 Induction in Islet Cells Results in Protection From Apoptosis and Improved In Vivo Function After Transplantation. Diabetes.

[68] Wang H., Lee S.S., Gao W., Czismadia E., McDaid J., Ollinger R., Soares M.P., Yamashita K., Bach F.H. (2005). Donor Treatment With Carbon Monoxide Can Yield Islet Allograft Survival and Tolerance. Diabetes.

