# Supplementary material for: Photo-Polymerization Damage Protection by Hydrogen Sulfide Donors for 3D-Cell Culture Systems Optimization
Source: Int J Mol Sci. 2021 Jun 5;22(11):6095. doi: 10.3390/ijms22116095 (PMC8201135; doi:10.3390/ijms22116095)
Supplement: Supplementary file 1 [file ijms-22-06095-s001.zip › ijms-1230171-supplementary/Supplementary Materials_/Supplementary materials.pdf]

# Supplementary Materials

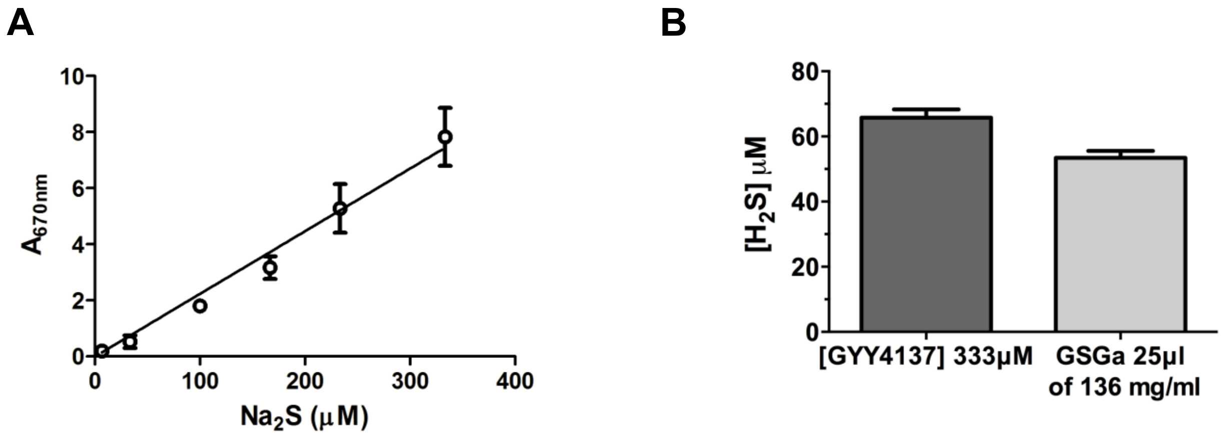


**Figure S1. Calibration curve of $H_2S$-release**. **A**) Calibration curve of $H_2S$ release obtained using different concentrations of $Na_2S$. **B**) Amount (in μM) of $H_2S$ released by 333 μM GYY4137 and 25 μl of GSGa (136 mg d.w./ml); measured by MB assay and spectrophotometric analysis.

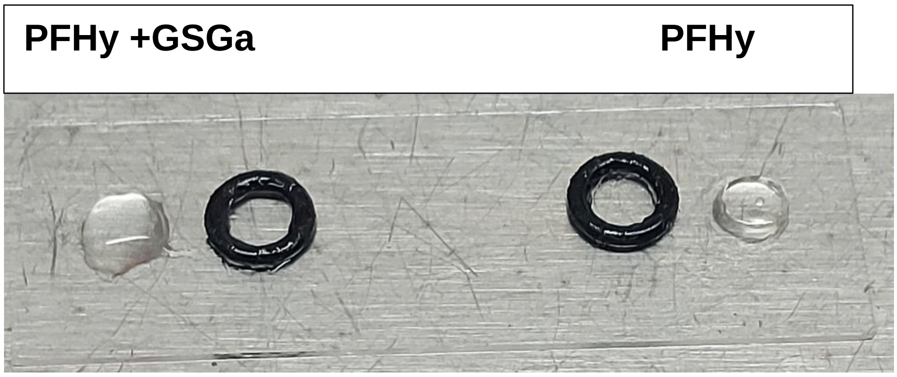


**Figure S2**. **Inhibition of the PFHy photopolymerization by GSGa.** PFHy (50μL) preparation in the presence and in the absence of GSGa (31.5 μg d.w./ 5μL).

**cMSC**

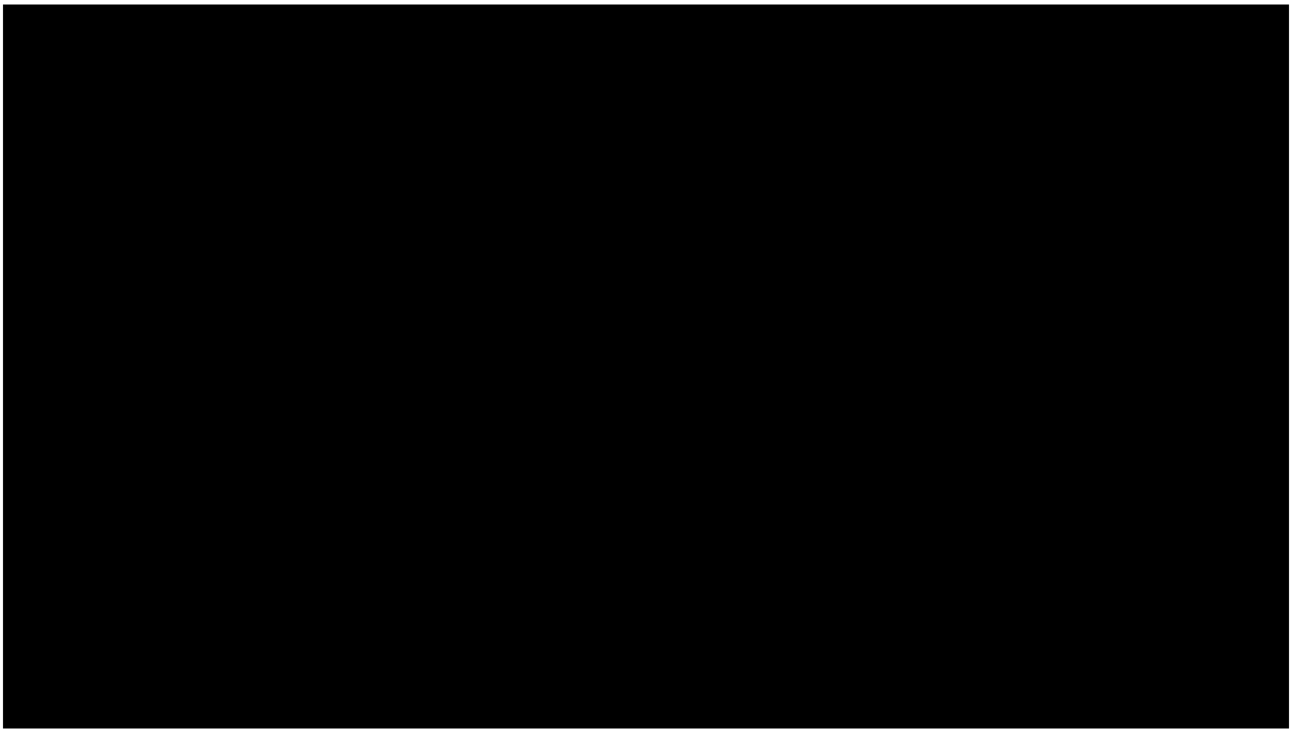

**GSGa-cMSC**

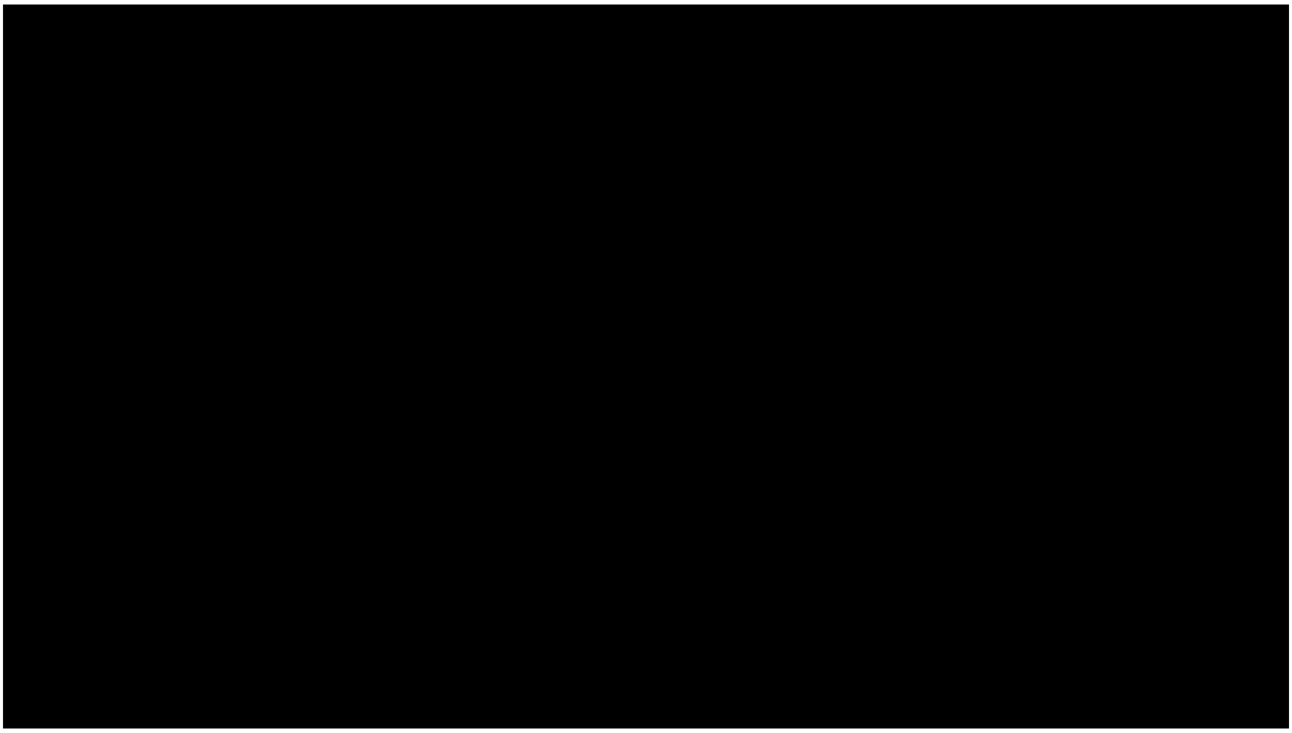

**Video of bright field microscopic analysis** of cMSC and GSGa pre-treated cMSC embedded in 3D-PFHy and cultured for 7 days.
